# Atmospheric Pressure Plasma Deposition of Hybrid Nanocomposite Coatings Containing TiO_2_ and Carbon-Based Nanomaterials

**DOI:** 10.3390/molecules28135131

**Published:** 2023-06-30

**Authors:** Regina Del Sole, Chiara Lo Porto, Sara Lotito, Chiara Ingrosso, Roberto Comparelli, Maria Lucia Curri, Gianni Barucca, Francesco Fracassi, Fabio Palumbo, Antonella Milella

**Affiliations:** 1 Dipartimento di Chimica, Università degli Studi di Bari Aldo Moro, 70125 Bari, Italysara.lotito@uniba.it (S.L.); lucia.curri@ba.ipcf.cnr.it (M.L.C.); francesco.fracassi@uniba.it (F.F.); antonella.milella@uniba.it (A.M.); 2 Istituto per i Processi Chimico Fisici, CNR, S.S. Bari, c/o Dipartimento di Chimica, Università degli Studi di Bari Aldo Moro, 70125 Bari, Italy; chiara.loporto@poliba.it (C.L.P.); c.ingrosso@ba.ipcf.cnr.it (C.I.); r.comparelli@ba.ipcf.cnr.it (R.C.); 3 Consorzio Interuniversitario Nazionale per la Scienza e Tecnologia dei Materiali INSTM, Unita di Ricerca di Bari, 70126 Bari, Italy; 4 Dipartimento di Scienze e Ingegneria della Materia, dell’Ambiente ed Urbanistica, Università Politecnica delle Marche, 60121 Ancona, Italy; g.barucca@staff.univpm.it; 5 Istituto di Nanotecnologia, CNR, S.S. Bari, c/o Dipartimento di Chimica, Università degli Studi di Bari Aldo Moro, 70125 Bari, Italy

**Keywords:** plasma deposition, nanocomposite coating, TiO_2_, photocatalysis, aerosol-assisted plasma, carbon nanomaterials

## Abstract

Among the different applications of TiO_2_, its use for the photocatalytic abatement of organic pollutants has been demonstrated particularly relevant. However, the wide band gap (3.2 eV), which requires UV irradiation for activation, and the fast electron-hole recombination rate of this n-type semiconductor limit its photocatalytic performance. A strategy to overcome these limitations relies on the realization of a nanocomposite that combines TiO_2_ nanoparticles with carbon-based nanomaterials, such as rGO (reduced graphene oxide) and fullerene (C_60_). On the other hand, the design and realization of coatings formed of such TiO_2_-based nanocomposite coatings are essential to make them suitable for their technological applications, including those in the environmental field. In this work, aerosol-assisted atmospheric pressure plasma deposition of nanocomposite coatings containing both TiO_2_ nanoparticles and carbon-based nanomaterials, as rGO or C_60_, in a siloxane matrix is reported. The chemical composition and morphology of the deposited films were investigated for the different types of prepared nanocomposites by means of FT-IR, FEG-SEM, and TEM analyses. The photocatalytic activity of the nanocomposite coatings was evaluated through monitoring the photodegradation of methylene blue (MB) as a model organic pollutant. Results demonstrate that the nanocomposite coatings embedding rGO or C_60_ show enhanced photocatalytic performance with respect to the TiO_2_ counterpart. In particular, TiO_2_/C_60_ nanocomposites allow to achieve 85% MB degradation upon 180 min of UV irradiation.

## 1. Introduction

The study of nanocomposite materials has received increasing attention in recent decades because their properties arise from the combination of their components, thus resulting superior to those of the single constituents. Nanomaterials such as metal nanoparticles, quantum dots, and carbon based nanostructures can be used as fillers in different polymeric matrices, thus leading to an enhancement of the mechanical, electrical, thermal, or chemical properties of the host matrix [1]. In particular, among inorganic nanomaterials, semiconductor nanoparticles have been extensively investigated, as they exhibit characteristics remarkably different from the corresponding bulk materials and, depending on the size, i.e., large surface-area-to-volume ratio, higher reactivity and a characteristic response to light irradiation that results in the photogeneration of charge carriers. Looking at the ensemble of these properties, semiconductor nanomaterials based on TiO_2_ appear definitely suitable for photocatalysis applications that include the degradation of organic pollutants in different matrices and result in, for instance, effective water treatment. However, for conveniently addressing these kinds of applications, the technological issues of the separation and subsequent reuse of the nanomaterials needs to be solved, thus highlighting the urge to immobilize them on a solid support. In this perspective, the preparation of nanocomposites featuring a polymeric matrix that serves as a host for immobilizing nanoparticles is found to be highly advantageous. On the other hand, the judicious choice of such host matrix plays an essential role, as it needs to be resistant to UV irradiation, durable, stable, and chemically inert, but also able to guarantee an effective interaction between the molecules of the organic pollutants to be degraded and the photocatalyst surface [2]. Deposition as thin films of nanocomposites obtained either via bare mixing of the components or via in situ polymerization, such as in the sol-gel method [3], can be achieved through various conventional methods [4], including spin coating [5], solution casting, hot pressing, dip coating, and melt intercalation [6]. 

Plasma technologies can be very convenient for fabricating functional nanocomposite coatings [7]. Recently, aerosol-assisted plasma deposition (AAPD) has been demonstrated achieve the successful deposition at atmospheric pressure of nanocomposite films onto solid supports. This approach is particularly valuable because it also allows thermo-degradable or scarcely volatile species to be embedded in a polymeric matrix starting from an aerosol of their solution or dispersion. Further advantages of AAPD, in comparison to other more conventional deposition techniques, can be identified in the reduced and controlled production of chemical waste during the process and in the possibility of easily depositing films virtually onto any kind of substrate; it is also heat sensitive, irrespective of its geometry and morphology. Indeed, the deposition of homogeneous films can be achieved in a one-step procedure, starting from a monomer or its solution, while nanocomposite coatings can be obtained from a suspension/solution of the nanofiller and the use of an auxiliary feed of the monomer as a gas or as an aerosol [8]. In particular, deposition onto different solid supports of nanocomposite coatings containing TiO_2_ nanoparticles in an organic polymeric matrix has been recently described for addressing photocatalytic applications [9]. 

Nowadays, TiO_2_ is one of the most commonly used semiconductor photocatalysts because of its strong oxidizing activity, superhydrophilicity, chemical stability, long durability, low toxicity, cost effectiveness, and transparency to visible light. The band gap is wide—3.2 eV for anatase, the most photoactive phase, and 3.0 eV for the rutile phase—and it is even more blue-shifted when nanoparticles are considered. Thus, UV irradiation of this material leads to the photogeneration of charge carriers (electron and holes), able to migrate to the photocatalyst surface and take part in redox reactions [10,11]. However, photocatalytic activity is limited by charge recombination phenomena that take place competitively inside the semiconductor material. The charge recombination rate can be reduced through combining the photocatalyst with noble metals [12,13], though this strategy leads to a consequent increase of the total cost of the system. A more feasible and convenient alternative is represented by coupling TiO_2_ with carbon nanomaterials (CNMs) such as carbon nanotubes [14,15], fullerenes (C_60_) [16], graphene [17], reduced graphene oxide (rGO) [18], carbon dots [19], carbon nanofibers [20], and graphene quantum dots [21]. Indeed, the combination of TiO_2_ with carbon-based materials could improve the transportation of photocarriers in the photocatalysis process via electronic interaction with TiO_2_, and, in addition, the delocalized conjugated structure present in these materials can promote an efficient photo-induced charge separation and limit charge recombination. Furthermore, TiO_2_-carbon based nanomaterial composites have been widely proven to exhibit photocatalytic activity under visible light, due to a possible band gap modification or sensitization effect [22]. However, this aspect will not be investigated herein. This work is focused on the preparation of TiO_2_-based nanocomposites coatings loaded, respectively, with rGO and C_60_. Indeed, rGO is characterized by a honeycomb structure, similar to that of graphene, also featuring domains with oxygenated functionalities able to enhance its dispersibility in polar solvents. Thanks to its high work function (4.42 eV), rGO coupled to TiO_2_ results in the transfer of photogenerated electrons from the conduction bands of TiO_2_ to rGO sheets. Once there, these electrons can be effectively stabilized by the sp^2^ carbon network, thus reducing charge recombination phenomena [23]. Similarly, C_60_, with its work function of 4.70 eV [24], acts as an electron trap, increasing the lifespan of electron–hole pairs [25]. Also, the feeding mixture for the AAPD process has been defined so as to generate an organic–inorganic hybrid matrix, suitable to the photocatalytic fillers and able to more effectively sustain photochemically induced degradation.

To the best of our knowledge, this is the first time that hybrid TiO_2_/rGO and TiO_2_/C_60_ nanocomposites are deposited via AAPD and their morphological and chemical features are correlated with their photocatalytic activity. Thus, we investigate their potential as a valuable option for water treatment technologies thanks to the great versatility of the deposition method, which enables the integration of photocatalytic coatings in photoreactors. 

## 2. Results and Discussion

### 2.1. Nanocomposite Coatings Characterization

**Figure 1:**
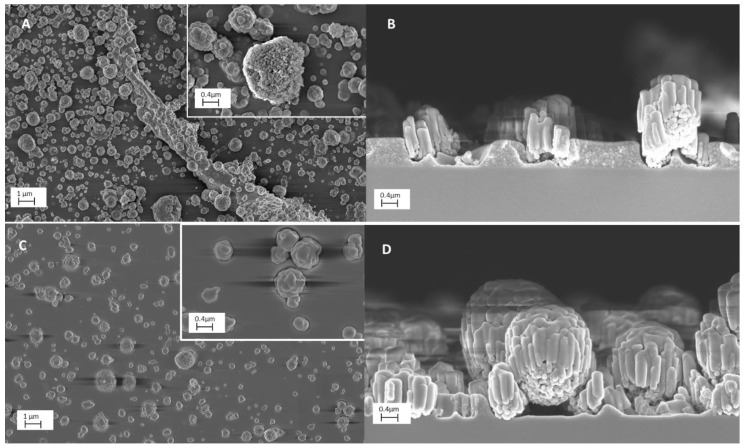
SEM images of *ncTiO*_2_*_rGO* and *ncTiO*_2_*_C*_60_ nanocomposites coatings. (**A**) Top view of *ncTiO*_2_*_rGO* at 10 kx and 100 kx (inset) magnification and (**B**) corresponding cross section at 50 kx magnification. (**C**) Top view of *ncTiO*_2_*_C*_60_ at 10 kx and 100 kx (inset) magnification and (**D**) corresponding cross section at 50 kx.

Figure 1 reports the top and cross section SEM images of the TiO_2_ composite samples containing rGO or C_60_, namely *ncTiO*_2_*_rGO* and *ncTiO*_2_*_C*_60_, respectively. Both samples are characterized by agglomerates incorporated into or protruding from a polymeric matrix. SEM images of rGO and C_60_ powders are reported for comparison in the Supplementary Materials (Figure S1). It can be observed that the density of the nanoparticles in the coating is higher for *ncTiO*_2_*_rGO* (1A) than for the *ncTiO*_2_*_C*_60_ nanocomposite (1C). In addition, in Figure 1A, it is worth noting a ribbon-like structure, possibly ascribable to folded rGO sheets. Cross section images (Figure 1B,D) highlight the occurrence of aggregates, consisting of submicrometric cylindrical structures for both types of samples. Such aggregates appear characterized by a more regular spheroidal shape in the case of *ncTiO*_2_*_C*_60_. Conversely, the CNM-free nanocomposite coating, *ncTiO*_2_, do not exhibit such features, as it can be observed in the SEM micrographs reported in Figure S2, in agreement with what was previously reported [9]. Hence, such submicrometric cylindrical features can likely be ascribed to the presence of CNM in the coating; however, further investigations would be needed to account for the different morphology observed upon the addition of rGO or C_60_ as fillers, respectively. 

In accordance with SEM side view images, profilometry analysis results, reported in Table 1, confirm that the *ncTiO*_2_*_C*_60_ nanocomposite is thicker than the *ncTiO*_2_*_rGO* one, and the thickness of the control sample (coating containing TiO_2_ without CNM) is intermediate between the two.

**Figure 2:**
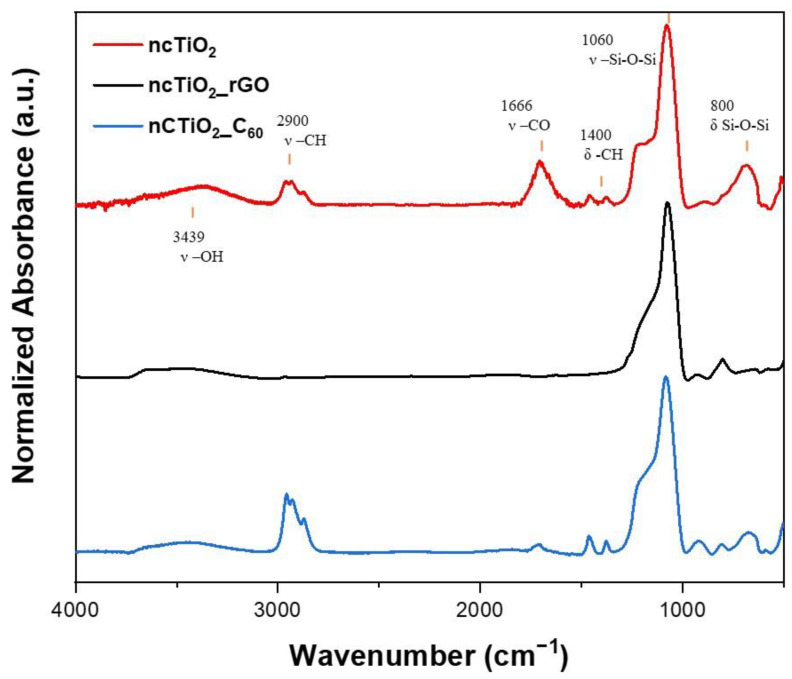
FT-IR spectra of *ncTiO*_2_ control (red line), *ncTiO*_2_*_rGO* (black line) and *ncTiO*_2_*_C*_60_ (blue line) samples.

FT-IR spectra of *ncTiO*_2_*_rGO* and *ncTiO*_2_*_C*_60_ samples, along with that of the TiO_2_-based reference sample, are shown in Figure 2. The signals of TiO_2_ and of CNM are likely hidden under the more intense signals of the matrix, thus preventing the retrieval of any insight on the fillers in the nanocomposite. On the other hand, the analysis allows us to elucidate the chemical composition of the matrix. The spectra of *ncTiO*_2_-(red line) and *ncTiO*_2_*_C*_60_-containing coatings (blue line) are characterized by peaks at 2900 cm^−1^, typical of C-H stretching (ν-CH) in sp^3^ hybridized carbon with corresponding bending at around 1400 cm^−1^ (δ-CH). Furthermore, the presence of a sharp signal at 1666 cm^−1^, ascribed to C=O stretching (ν-CO), could indicate the formation of coordination complexes of Ti with carbonyl compounds, as reported in the literature [26]. On the contrary, the IR spectrum of the *ncTiO*_2_*_rGO* nanocomposite sample (black line) points to the presence of an inorganic siloxane matrix. Furthermore, the spectra of all the samples are characterized by a sharp signal at 1060 cm^−1^ due to Si-O-Si stretching mode (ν-Si-O-Si) [27], a band at 800 cm^−1^ ascribed to Si-O-Si bending vibration (δ-Si-O-Si) likely superimposing the vibrational modes of TiO_2_ network [28], and a weak signal at 3459 cm^−1^ accounting for the -OH stretching (ν-OH) of polysiloxane spectra profiles.

The FT-IR results on the organic/inorganic character of the matrix in the nanocomposite coatings are confirmed via the EDX analysis, reported in Table 2. Indeed, it is worth noticing that the lowest C/Si value, accounting for a more inorganic nature of the coating, is found for *ncTiO*_2_*_rGO* (0.07), increasing in the *ncTiO*_2_ control sample (0.85) and going up to 1.82 for the *ncTiO*_2_*_C*_60_ nanocomposite. More information on the titanium content in the nanocomposite is provided via evaluation of the Ti atomic percentage and Ti/Si values. Higher Ti content is found in the sample *ncTiO*_2_*_rGO* (Ti = 3% and Ti/Si ratio = 0.05), while it decreases in the bare TiO_2_ reference sample (Ti = 1.7% and Ti/Si ratio = 0.04) and further reduces in *ncTiO*_2_*_C*_60_ nanocomposite (Ti = 0.5% and Ti/Si ratio = 0.02). These results are also consistent with the features observed in the SEM micrographs of the sample, which show a higher aggregate density in the *ncTiO*_2_*_rGO* nanocomposite than the *ncTiO*_2_*_C*_60_ sample. 

**Figure 3:**
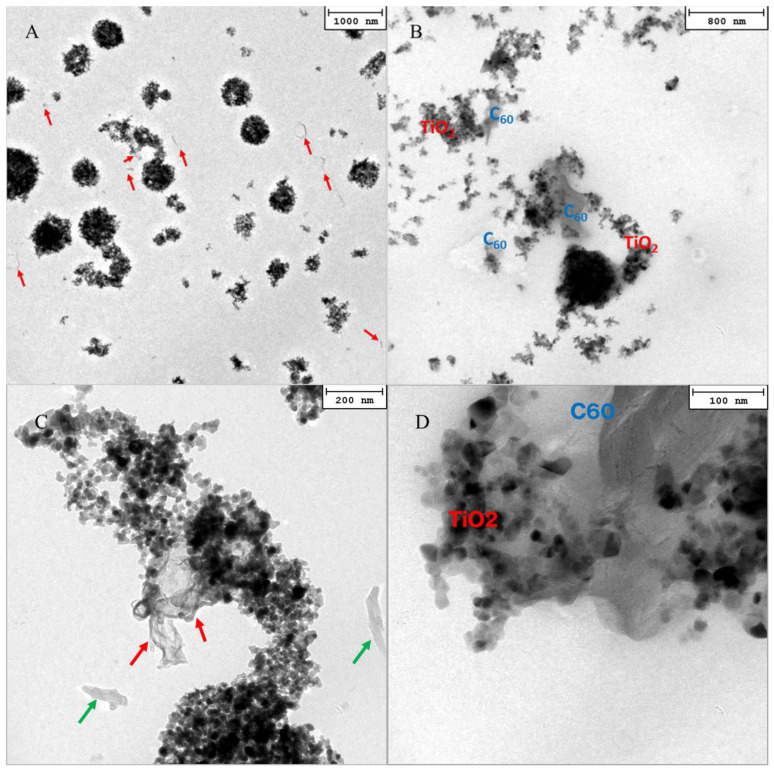
Bright field TEM images of *ncTiO_2__rGO* (**A**,**C**) and *ncTiO_2__C*_60_ (**B**,**D**). Green and red arrows indicate isolated and TiO_2_-coupled rGO sheets, respectively.

TEM micrographs of the nanocomposite coatings are shown in Figure 3. TiO_2_ nanoparticles are clearly evident in the images due to their high atomic number contrast and tend to form aggregates of different sizes and shapes. In particular, Figure 3A reveals how TiO_2_ agglomerates are uniformly distributed in the polymeric matrix of the *ncTiO*_2_*_rGO* coating. The rGO sheets are more difficult to identify in the image due to their lighter contrast with respect the TiO_2_ particles; therefore, the features ascribed to these structures are pointed out with arrows in the micrographs. The observations, performed at different magnifications, indicate a homogeneous distribution of the rGO sheets that are found both in contact with TiO_2_ nanoparticles (red arrows) and isolated in the polymeric matrix (green arrows), as shown in Figure 3C. The distribution of TiO_2_ nanoparticles within the *ncTiO*_2_*_C*_60_ coating is shown in Figure 3B and at higher magnification in Figure 3D. *C*_60_ gives rise to large structures that can be identified in the TEM images more easily than the rGO sheets. As in the *ncTiO*_2_*_rGO* coating, also in this sample, a uniform distribution of TiO_2_ and C_60_ agglomerates is clearly evident, although, interestingly, C_60_ nanomaterial appears, in general, to share more contact area with the TiO_2_ nanoparticles. The nature of the TiO_2_ nanoparticles and rGO/C_60_ carbon nanomaterials was further investigated via selected area electron diffraction (SAED) and high-resolution (HR) TEM analyses (Figure S3). In particular, SAED measurements indicate an anatase structure for the TiO_2_ nanoparticles and their good crystallinity in the nanocomposite (Figure S3A,B), and HRTEM observations demonstrate the effective presence of rGO sheets (Figure S3C) and C_60_ structures (Figure S3D) in the deposited coatings.

### 2.2. Photocatalytic Activity Evaluation

**Figure 4:**
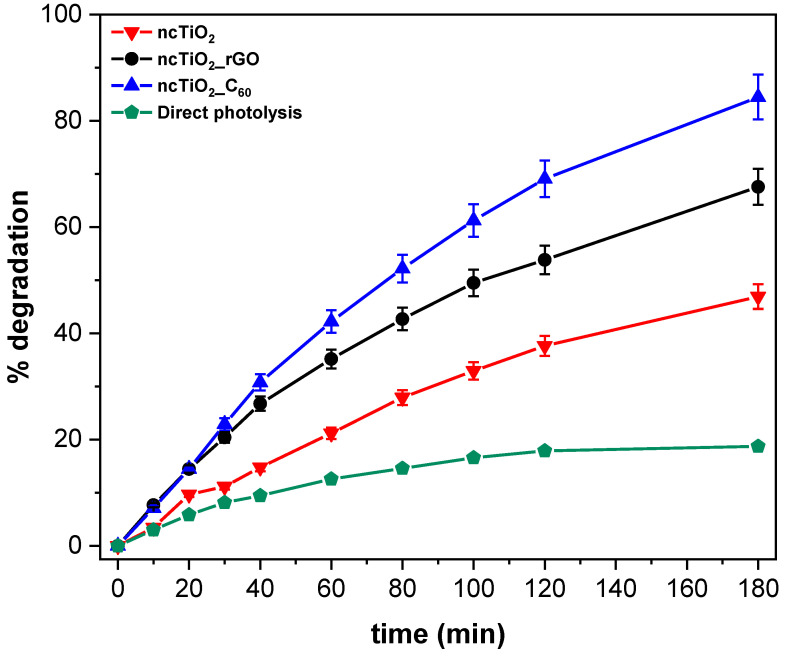
Time course of MB photodegradation reaction assisted by *ncTiO*_2_*_C*_60_ nanocomposite, *ncTiO*_2__*rGO* nanocomposite in siloxane matrix, and *ncTiO*_2_ nanocomposite coatings deposited on glass and of bare glass (direct photolysis). The reported data are referred to as mean values ± standard deviation obtained from the analysis of three replicates.

The photodegradation curves of MB are reported in Figure 4. The results highlight an enhancement in photocatalytic activity in the nanocomposite incorporating CNM besides TiO_2_ in the siloxane matrix, when compared to the TiO_2_-only-based counterpart. In the experiment assisted by the *ncTiO*_2_*_rGO* film (black line), 68% degradation of the model pollutant is observed after 180 min of irradiation, while in the case of the *ncTiO*_2_*_C*_60_ nanocomposite, an even better performance is recorded (blue line), reaching an MB degradation value of 85%. The rate of MB photodegradation by the *ncTiO*_2_ control sample (red line) is 47% after 180 min of irradiation, significantly lower than the result recorded for the CNM-TiO_2_-based nanocomposites. Direct photolysis (green line) does not exceed 20% of MB degradation, suggesting its negligible contribution to the degradation process assisted by the prepared photocatalytic nanocomposites.

In Table 3, a summary of the highest degradation value obtained after irradiating the dye solution for 180 min, the kinetic constant (k) of the process, and the related R^2^ are reported. Remarkably, reactions assisted by TiO_2_/CNM-based nanocomposites show a kinetic constant higher than that observed when the reference sample, *ncTiO*_2_, is used. In particular, the kinetic constant is twice as high when rGO is also present in the TiO_2_ nanocomposite coating, and is nearly three time higher in the C_60_-based counterpart. In addition, two further control samples, prepared in the same deposition conditions, but without TiO_2,_ were also tested to evaluate the intrinsic photocatalytic activity of each CNM nanocomposite coating. The results reported in Table 3 point out that the nanocomposite sample embedding C_60_ alone achieved an MB photodegradation rate of 44%, a value slightly lower that that achieved with the TiO_2_ control itself. Indeed, the intrinsic photocatalytic activity of C_60_ has already been demonstrated in the literature, though in water suspension [29,30]. On the other hand, the rGO-based nanocomposite shows no intrinsic photocatalytic activity. However, the enhancement in the photocatalytic activity of the *ncTiO*_2_*_C*_60_ nanocomposite cannot be ascribed just to the presence of a higher amount of photocatalytic species (TiO_2_ and C_60_) in the coating. Indeed, C_60_ has been reported to be able not only to promote photocatalysis itself but also to act as an efficient co-catalyst when combined in nanocomposites with TiO_2_, thus enhancing the performance of the whole composite material.

**Figure 5:**
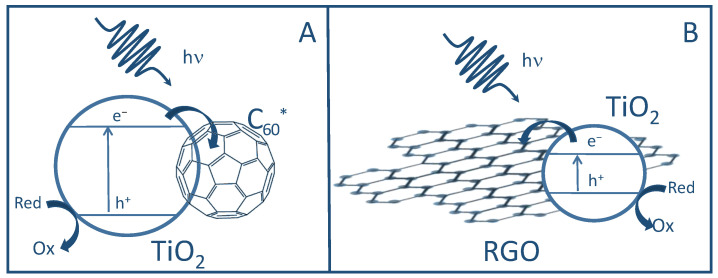
Sketches of the oxidation process assisted by TiO_2_/C_60_ (**A**) and TiO_2_/RGO (**B**).

C_60_ strongly absorbs in the visible range and moderately in the UV range (with a band gap for solid C_60_ of 1.6–1.9 eV) and, under irradiation, can form two excited states: a transient singlet (^1^C_60_*) and a longer-lasting triplet (^3^C_60_*) [21,30]. These excited states can easily act as electron acceptors (forming the anion C_60_^−^) and scavenge the electrons photogenerated in TiO_2,_ thus increasing the charge separation, lowering the occurrence of recombination, and increasing the photocatalytic activity of pristine TiO_2_ (Figure 5). In fact, they can also act as electron donors, sensitizing TiO_2_ injecting electrons in its CB. On the other hand, the intrinsic photocatalytic activity of C_60_ can be accounted for by its ability to photo-generate electrons. Such a two-fold role in transferring electrons is quite peculiar and explains the strong increase in the activity of the composite nanomaterial [31]. On the other hand, when RGO is combined with TiO_2_, it can limit the recombination of photogenerated charges due to its electron mobility, thus acting as an electron sink (Figure 5).

The enhancement obtained for the nanocomposite containing C_60_, which is higher than that observed for the *ncTiO*_2_*_rGO*, can be also accounted for through considering the morphology of the nanocomposites and the interactions occurring among the nanofillers therein. In *ncTiO*_2_*_C*_60_, the nanofiller aggregates are more exposed, being less immersed in the matrix, and, thus, they present a larger surface available for interacting with the MB molecules, which leads to a higher degradation extent.

Moreover, the morphological investigation of the prepared samples highlights that interactions between TiO_2_ nanoparticles and C_60_ in *ncTiO*_2_*_C*_60_ are larger than that those observed between TiO_2_ and RGO, thus suggesting a more efficient electron scavenging effect [32] which limits charge recombination in the photocatalyst. Such a feature, along with the more inorganic nature of the host matrix observed in this sample, could account for the higher photocatalytic activity.

Furthermore, the different electronic properties of the two CNMs may play a role, since rGO has high electric conductivity [33], while C_60_ shows high resistivity [34] that could turn in a different oxidation extent of the matrix during the deposition process.

While these factors may account for the different performance of the rGO- and C_60_-based TiO_2_-containing nanocomposites, their complex interplay and the complexity of the plasma system, involving the concomitant participation of different compounds in the deposition process, still deserves a deeper investigation to fully elucidate the structure–function relationship and, thus, entirely explain the photocatalytic behaviour of the prepared nanocomposites.

## 3. Materials and Methods

### 3.1. Suspensions Preparation

TiO_2_ P25 Aeroxide (Evonik, Essen, Germany) was suspended in a mixture of hexamethyldisiloxane (HMDSO, ≥98% Sigma Aldrich, Darmstadt, Germany) and isopropyl alcohol (IPA, Honeywell, ≥99.8% Charlotte, NC, USA) (10/90 *v*/*v*) at a concentration of 10 mg/mL. Reduced graphene oxide (highly porous rGO, Graphene Supermarket, New York, NY, USA) and fullerene C_60_ (99.4%, Italy Nanocage S.R.L.) were individually dispersed at a concentration of 1 mg/mL in distilled deionized water. The suspensions were sonicated in an ultrasonic bath (CEIA, mod. CP102) for 1 h. 

### 3.2. Plasma Deposition of Nanocomposite Coatings

An in-house-built dielectric barrier discharge reactor was used in order to perform AAPD, as in previous works [8,35]. Briefly, the core of the reactor was a plexiglass chamber hosting two parallel silver-coated alumina electrodes (5 × 8 cm^2^ wide and 0.63 mm thick), separated by a 2 mm gap. The reactor chamber was connected to two distinct pneumatic atomizers (mod. 3076, TSI): the former was fed with the TiO_2_ suspension in IPA/HMDSO (90/10 *v*/*v*), *aerosol 1*, supplying a 2.5 slm He flow, while the latter was fed with the suspension of carbon nanomaterial (containing rGO or C_60_), *aerosol 2*, admitted with a 3.5 slm He flow. Also, a nanocomposite coating containing TiO_2_ only was deposited as a reference (*ncTiO*_2_). The aerosol reached the electrodes area through a slit and was evacuated by means of an aspirator located on the opposite side. The plasma discharge was ignited between the two electrodes in continuous mode using a wideband AC power amplifier (Al-1000-HF-A by AMP-LINE corp., West Nyack, NY, USA) driven by a function generator (Model TG-1000 by TTi). The amplifier was connected to the high-voltage electrode with an HV transformer (Model AL-T1000, AMP-LINE corp.). The electrical characteristics of the plasma were investigated, measuring the voltage and the current delivered to the system with a high-voltage (P6015A, Tektronix, Beaverton, OR, USA) probe and a resistance-type current probe, both connected to an oscilloscope (TDS 2014C, Tektronix, Beaverton, OR, USA). A peak-to-peak voltage of 6 kV and a frequency of 24 kHz were applied to generate plasma (total power density 1.9 W/cm^2^), and the deposition time was set at 15 min. Slices of P-doped Si wafer (1 cm × 1 cm) were used as solid substrates for the nanocomposite coating characterization, while microscope glass slides (1.5 cm × 1.5 cm) were chosen for the photocatalysis test. Deposition conditions are summarized in Table 4. 

### 3.3. Nanocomposite Coatings Characterization

The morphology of the coatings was investigated by means of field emission gun—scanning electron microscopy (FEG-SEM) carried out with a Zeiss Supra 40 SEM equipped with a Gemini field-effect emission gun. The extraction voltage was set to 3 kV, and the brightness, contrast, and working distance (varying in the range of 2–4 mm) were optimized for each acquisition. Also, images of the bare rGO and C_60_ powders, gently pressed onto conductive double-sided adhesive tape, were acquired preliminarily. 

Energy-dispersive X-ray spectroscopy analysis (EDX) was carried out to determine the chemical composition of the coating in terms of Ti atomic percentage (%Ti) and Ti/Si and C/Si ratios in order to estimate, respectively, the content of Ti, its relative contribution with respect to the matrix represented by the Si content, and the organic/inorganic character of the polymeric matrix itself (C/Si). The analysis was carried out by means of an INCA Oxford microanalysis probe mounted onto the Zeiss Supra 40 SEM. 

Further chemical information was retrieved thanks to Fourier-transform infrared spectroscopy (FT-IR): spectra (32 scans per analysis at a 4 cm^−1^ resolution) were recorded in transmission mode with a Vertex 70v Bruker spectrometer. The spectrometer was evacuated to less than 150 Pa for 5 min before each acquisition. The spectra were normalized to the maximum intensity of the Si-O-Si stretching band at 1060 cm^−1^. 

The thickness of the coatings was measured by means of a KLA-Tencor (Milpitas, CA, USA) D-120 profilometer using polished silicon chips as substrates and scratching part of the coating with a scalpel.

Finally, to confirm the embedding of carbon nanomaterials (rGO and C_60_ powders) and TiO_2_ nanoparticles in the deposited coatings, to investigate their spatial distribution within the matrix, and to determine possible occurrence of a contact interface between TiO_2_ particles and CNM, transmission electron microscopy (TEM) analysis was performed using a Philips CM200 microscope operating at 200 kV and equipped with a LaB_6_ filament. For observations, the coatings were directly deposited on conventional carbon-coated transmission electron microscopy (TEM) grids (Carbon covered copper grids, 300 µm mesh, Electron Microscopy Sciences, Hatfield, PA, USA) using the same conditions described in Table 1, except for the deposition time, which was shortened to 30 s in order to obtain coatings with a thickness suitable for TEM analysis.

### 3.4. Photocatalytic Activity Evaluation

The photocatalytic activity of the nanocomposite coatings was evaluated through monitoring the discoloration of a model pollutant solution by means of a UV-vis spectrophotometer (Cary 60 UV-Vis, by Agilent, Santa Clara, CA, USA). Spectra were collected in slow mode in the 450–800 nm range in polystyrene semi-micro cuvettes: methylene blue (MB) (Sigma Aldrich, Darmstadt, Germany) was chosen as the target molecule. Samples prepared on microscope slide glasses were placed in the bottom of a 50 mL beaker. Next, 5 mL of MB 10^−5^ M solution was poured in the beaker under magnetic stirring and left in the dark for 15 min as a conditioning step. Absorbance at 665 nm, corresponding to the characteristic peak of MB, was measured for the prepared solution after the conditioning step to isolate the contribution of the adsorption of MB on the photocatalyst surface due to the discoloration process. Then, the system was irradiated from above by means of two germicidal UV lamps (HNS 15 W G13, OSRAM, λ > 200 nm). Absorbance at 665 nm was measured at defined intervals of time up to 180 min, and the degradation percentage, that here is assumed to be correlated to the discoloration [36], was calculated using the following equation:

$$\%degradation\;of\;MB=\left[100-\left(\frac{Abs_{t}*100}{Abs_{t_{0}}}\right)\right]$$

where *Abs_t_*_0_ is the absorbance measured after the conditioning step and *Abs_t_* is the absorbance value at a given time. The absorbance values up to 40 min were used to calculate the kinetic constant (*k*) as the slope of the linear fit of the *ln(C*_0_/*C_t_)* vs. *t* graph. The *R*^2^ of the linear fit was also calculated. The same procedure was then repeated in dark conditions, and for all samples, the *%degradation of MB* was found to be negligible, thus indicating only a limited adsorption of MB on the coatings.

## 4. Conclusions

Aerosol-assisted atmospheric pressure plasma deposition is confirmed as a valuable technique for the deposition of nanocomposite coatings. The aerosol-assisted plasma deposition of nanocomposite coating containing two distinct nanofillers has been investigated here for the first time, to the best of the authors’ knowledge. After depositing hybrid nanocomposite siloxane-based films containing TiO_2_ and two different types of nanofillers, rGO or C_60_, respectively, their thorough investigation has been performed. Nanocomposites containing TiO_2_ and rGO are thinner (<1 µm thick) and are characterized by a more inorganic matrix and a higher percentage of Ti than those loaded with TiO_2_/C_60._

The morphology of the *ncTiO*_2_*_rGO* coatings presents a density of aggregates higher than that found for *ncTiO*_2_*_C*_60_. In the case of *ncTiO*_2_*_C*_60,_ the aggregates are more spheroidal and less immersed in the polymeric matrix. Furthermore, the *ncTiO*_2_*_C*_60_ nanocomposite presents a higher contact interface between the TiO_2_ and C_60_, probably also due to the geometrical characteristics of the carbon-based filler. As in *ncTiO*_2_*_rGO*, rGO sheets have been found to interact with TiO_2_ to a much lower extent, being, instead, found mostly isolated in the matrix.

Both the *ncTiO*_2_*_rGO* and *ncTiO*_2_*_C*_60_ nanocomposites have demonstrated enhanced photocatalytic performance during MB photodegradation with respect to the TiO_2_-based coatings. In particular, the *ncTiO*_2_*_C*_60_ nanocomposites already reach 85% MB degradation after 180 min of UV irradiation.

The proposed approach appears promising for photocatalytic applications and shows great potential in water remediation, as being able to plasma-deposit nanocomposite coatings on any kind of substrates enables their integration in photoreactors and, in turn, represents a technologically viable solution for water purification.

## Figures and Tables

**Table 1:** Thickness of the nanocomposite coatings deposited on Si wafer.

Sample	Thickness
*ncTiO*_2_	1210 ± 280 nm
*ncTiO*_2_ *_rGO*	870 ± 140 nm
*ncTiO*_2_ *_C*_60_	1690 ± 90 nm

**Table 2:** EDX analysis results for *ncTiO*_2,_ *ncTiO*_2_*_rGO* and *ncTiO*_2_*_C*_60_ nanocomposites.

Sample	%Ti	Ti/Si	C/Si
*ncTiO*_2_	1.7	0.04	0.85
*ncTiO*_2_ *_rGO*	3.0	0.05	0.07
*ncTiO*_2_ *_C*_60_	0.5	0.02	1.82

**Table 3:** MB degradation percentage (MB deg) at 180 min and kinetic constant (k) of the coatings.

Sample	MB Deg (%)	k (min^−1^)	R^2^
*ncTiO_2_*	47 ± 2	0.0041	0.99
*ncTiO_2__rGO*	68 ± 3	0.0078	0.99
*ncTiO_2__C*_60_	85 ± 4	0.0109	0.98
rGO nanocomposite (TiO_2_-free)	23 ± 1	0.0021	0.99
C_60_ nanocomposite (TiO_2_-free)	44 ± 2	0.0060	0.99
Direct photolysis	19 ± 1	0.0029	0.99

**Table 4:** Plasma deposition conditions.

Sample	Aerosol 1	Aerosol 2
*ncTiO*_2*_*_ *rGO*	TiO_2_ (10 mg/mL) IPA/HMDSO (90/10 *v*/*v*) He 2.5 slm	rGO (1 mg/mL) DDW He 3.5 slm
*ncTiO_2__C*_60_	TiO_2_ (10 mg/mL) IPA/HMDSO (90/10 *v*/*v*) He 2.5 slm	C_60_ (1 mg/mL) DDW He 3.5 slm
*ncTiO*_2_	TiO_2_ (10 mg/mL) IPA/HMDSO (90/10 *v*/*v*)	DDW He 3.5 slm

## Data Availability

The data presented in this study are available in this article and its Supplementary Materials.

## Supplementary Materials

The following supporting information can be downloaded at: https://www.mdpi.com/article/10.3390/molecules28135131/s1, Figure S1. SEM images at 10 kX of (A) rGO powder and (B) C_60_ powder. Figure S2. SEM images of ncTiO_2_ nanocomposite in siloxane matrix 10 kX top. Figure S3. (A) Bright field TEM image of TiO_2_ nanoparticles in the nanocomposite *ncTiO*_2__*rGO*; (B) corresponding selected area electron diffraction (SAED) pattern, all the diffraction spots can be associated to the TiO_2_ anatase phase; (C) High Resolution TEM micrograph of rGO sheets in the nanocomposite *ncTiO*_2__*rGO* (atomic planes are visible and univocally associated to rGO, inset); (D) High Resolution TEM micrograph of C_60_ structure in the nanocomposite *ncTiO*_2__*C*_60_ (atomic planes are visible and univocally associated to C_60_ structure, inset).
