# Supplementary material for: Atmospheric Pressure Plasma Deposition of Hybrid Nanocomposite Coatings Containing TiO2 and Carbon-Based Nanomaterials
Source: Molecules. 2023 Jun 30;28(13):5131. doi: 10.3390/molecules28135131 (PMC10343702; doi:10.3390/molecules28135131)
Supplement: Supplementary file 1 [file molecules-28-05131-s001.zip › molecules-2462781-supplementary.pdf]

Supporting Information

In Figure S1 SEM images are reported for rGO powder (A) and fullerene C_{60} powder (B). In Figure S1A, rGO sheets can be observed, while in S1B, the microstructure of C_{60} grains is displayed.

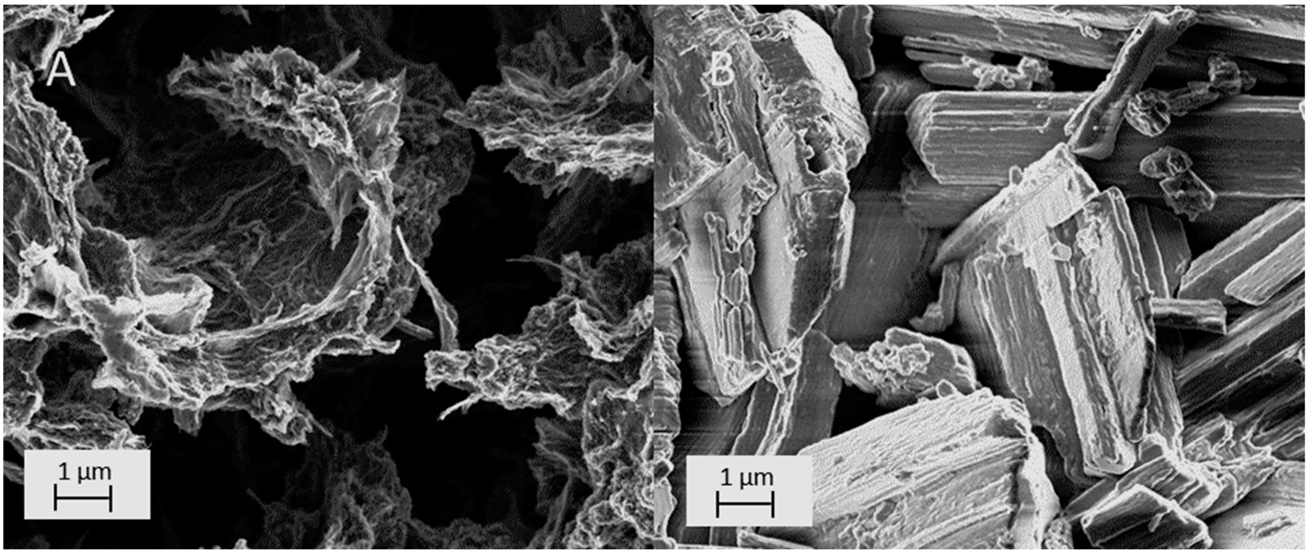

Figure S1. SEM images at 10kX of **(A)** rGO powder and **(B)** C_{60} powder.

In Figure S2 the SEM image at 10kX of *ncTiO2* is reported. It is possible to distinguish the TiO_2 particles, of spherical shape, but none of the morphological features retrieved in the *ncTiO2_rGO* and *ncTiO2_C60* are observable.

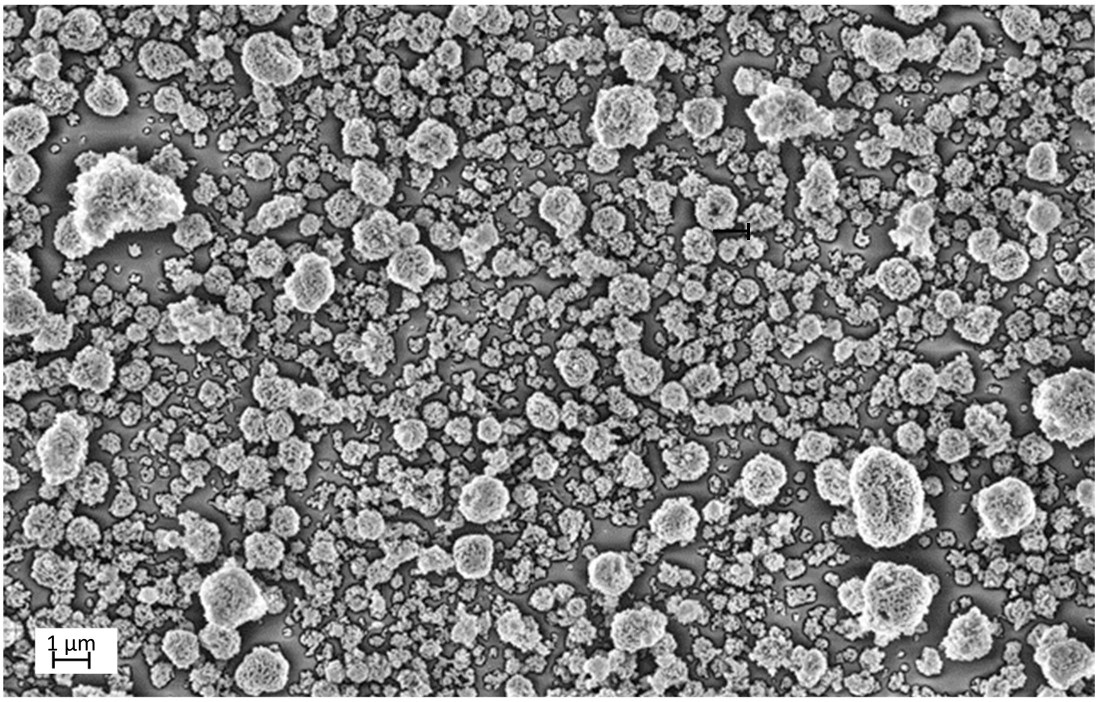

Figure S2. SEM images of $ncTiO_2$ nanocomposite in siloxane matrix 10kX top.

In Figure S3 HR-TEM micrograph for TiO_2 nanoparticles in the nanocomposite *ncTiO₂_rGO* along with the diffraction images are reported (S3A-S3B). TiO_2 nanoparticles are well crystalized and from HR-TEM micrographs the atomic planes are clearly visible and present on the whole nanoparticle. The calculated TiO_2 phase is anatase. In HR-TEM image for rGO (S3C), the atomic planes associated to the c axis can be observed, along with (002) planes (d=0.34nm), thus confirming the chemical nature of the structures in the micrographs. Eventually, in HR-TEM image (S3D), the atomic planes associated to the crystalline structure of buckminsterfullerene can be observed, confirming the presence of C_{60} in the nanocomposite.

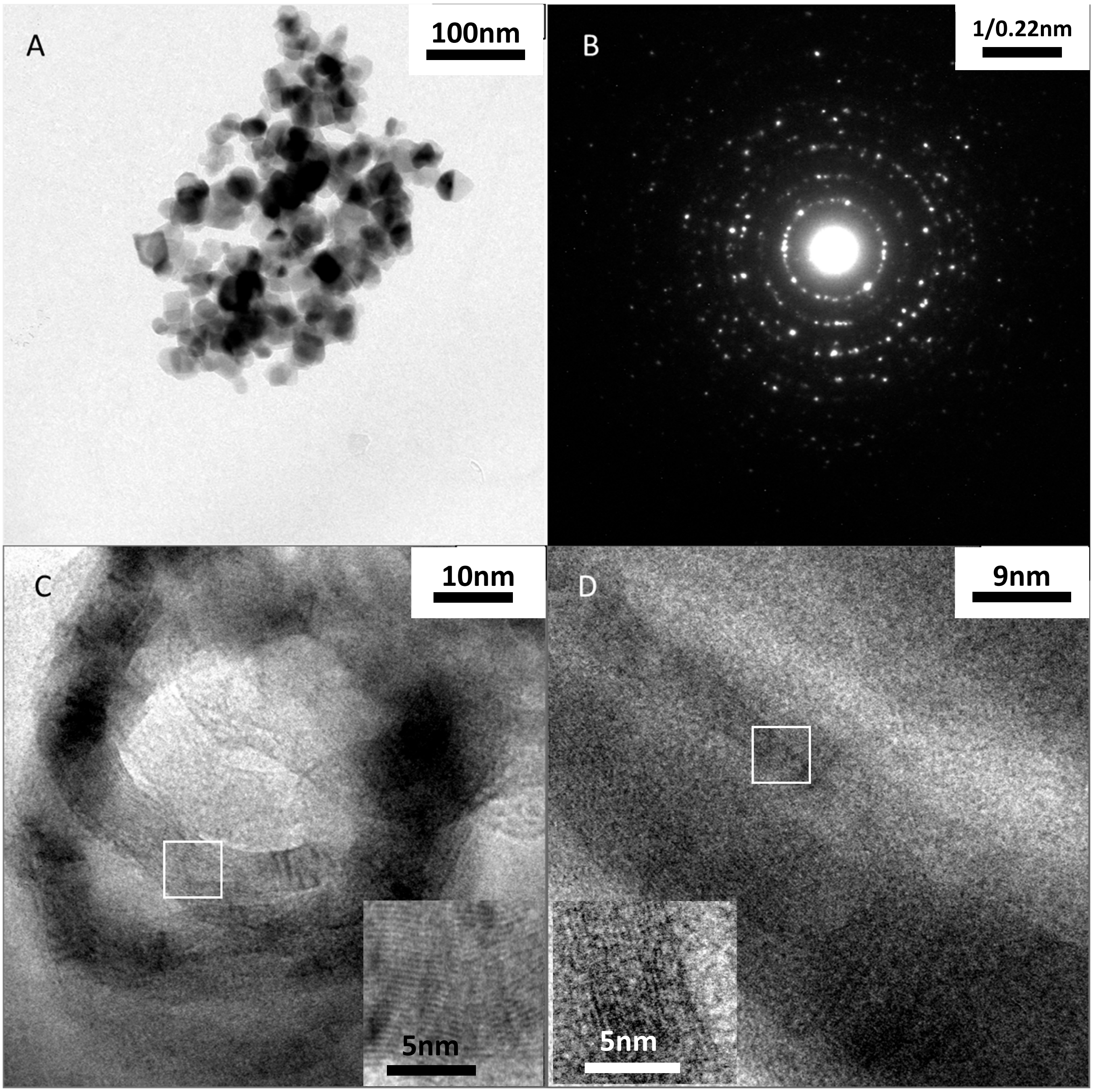

Figure S3. (A) Bright field TEM image of TiO_2 nanoparticles in the nanocomposite $ncTiO_2$_rGO; **(B)** corresponding selected area electron diffraction (SAED) pattern, all the diffraction spots can be

associated to the TiO_2 anatase phase; **(C)** High Resolution TEM micrograph of rGO sheets in the nanocomposite $ncTiO_2$_rGO (atomic planes are visible and univocally associated to rGO, inset); **(D)** High Resolution TEM micrograph of C_{60} structure in the nanocomposite $ncTiO_2$_C_{60} (atomic planes are visible and univocally associated to C_{60} structure, inset).
